# Climate-driven convergent evolution in riparian ecosystems on sky islands

**DOI:** 10.1038/s41598-023-29564-2

**Published:** 2023-02-16

**Authors:** S. J. Love, J. A. Schweitzer, J. K. Bailey

**Affiliations:** Department of Ecology and Evolutionary Biology, University of Tennessee, Knoxville, Dabney Hall, 1416 Circle Dr, Knoxville, TN 37996 USA

**Keywords:** Evolutionary ecology, Riparian ecology, Climate-change ecology, Ecological genetics, Forest ecology

## Abstract

Climate-induced evolution will determine population persistence in a changing world. However, finding natural systems in which to study these responses has been a barrier to estimating the impact of global change on a broad scale. We propose that isolated sky islands (SI) and adjacent mountain chains (MC) are natural laboratories for studying long-term and contemporary climatic pressures on natural populations. We used greenhouse common garden trees to test whether populations on SI exposed to hot and dry climates since the end of the Pleistocene have phenotypically diverged from populations on MC, and if SI populations have converged in these traits. We show: (1) populations of *Populus angustifolia* from SI have diverged from MC, and converged across SI, in reproductive and productivity traits, (2) these traits (cloning and aboveground biomass, respectively) are significantly correlated, suggesting a genetic linkage between them, and (3) the trait variation is driven by both natural selection and genetic drift. These shifts represent potentially beneficial phenotypes for population persistence in a changing world. These results suggest that the SI–MC comparison is a natural laboratory, as well as a predictive framework, for studying long-term responses to climate change across the globe.

## Introduction

Identifying how dominant terrestrial species have adapted in response to climate change is fundamental to understanding which traits are most important for population persistence and subsequent ecosystem functioning under contemporary climate change. Recent studies have shown that atmospheric climate gradients shape the linkage and feedback between genetically-based phenotypic variation and ecosystem functions at sub-continental scales^1–4^. This includes alterations to soil nutrient pools and bud break phenology driven by mean annual temperature and precipitation, elevation, and genetically-based soil conditioning^1,2,4^, as well as genetic divergence at the provenance level in atmosphere-plant-ecosystem feedback^3^. While these studies show that long-lived species evolve along contemporary landscape-scale abiotic environmental gradients to affect ecosystem function, they do not directly address how climate change has driven evolutionary dynamics, or which traits have been important for population persistence and ecosystem functioning. Evolution can occur on rapid timescales and is critical to understand in a time of rapid environmental change^5–8^. While multiple empirical approaches have been used to understand how species have and continue to respond to global climate variation^5,9–11^, approaches that address how species have evolved in direct response to climate change are still underdeveloped.

**Figure 1:**
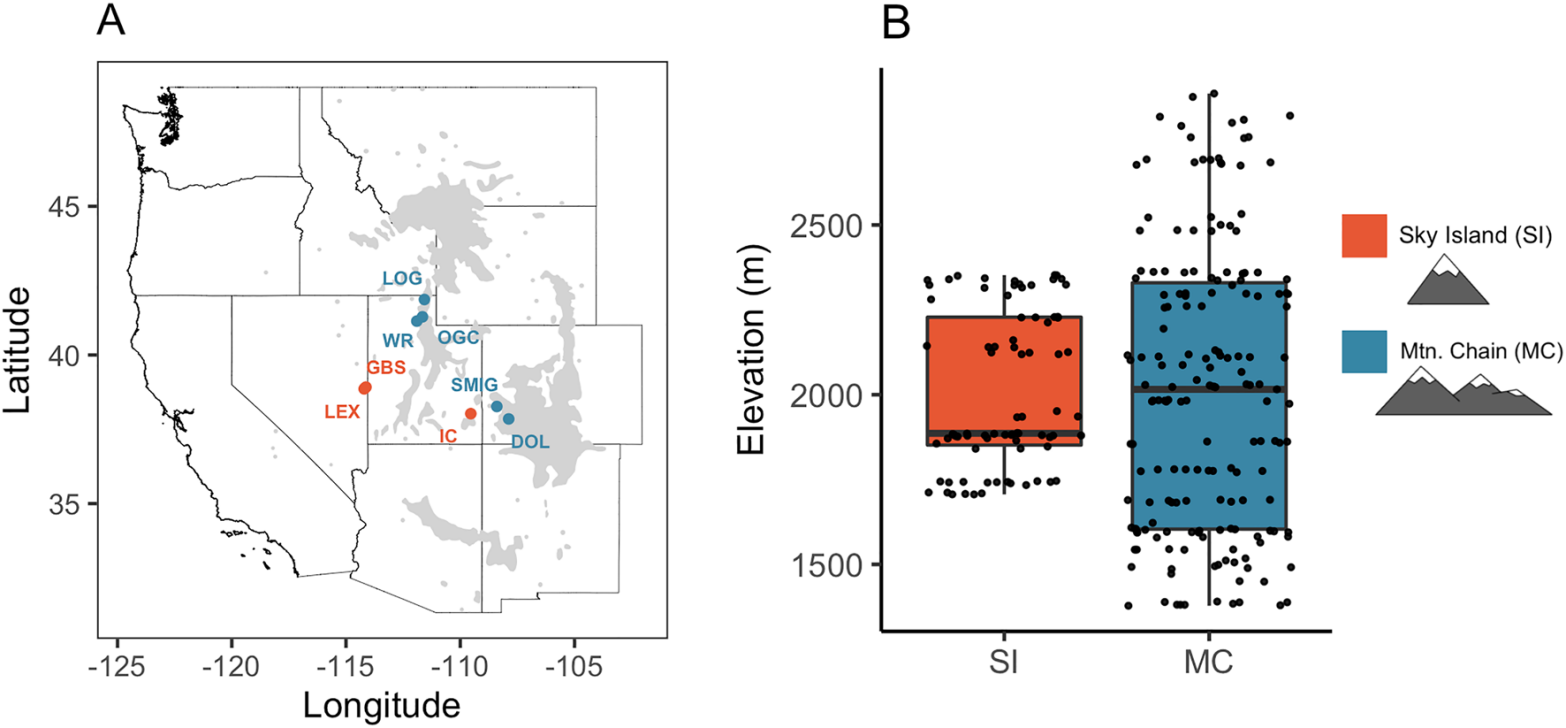
Study sites are distributed across the natural range of *Populus angustifolia* and do not differ in elevational range. (**A**). Map of riparian study sites across the intermountain western U.S. Sky islands (SI) are indicated in orange, n = 3 (IC, Indian Creek, UT; LEX, Lexington Creek, NV; GBS, Great Basin, NV), and mountain chains (MC) are indicated in blue, n = 5 (DOL, Dolores River, CO; LOG, Logan River, UT; OGC, Ogden Canyon, UT; SMIG, San Miguel River, CO; WR, Weber River, UT). The *Populus angustifolia* natural range is shaded in grey. (**B**). The average sampling elevation across the study sites is not significantly different between SI and MC, showing that environmental variation is not determined by range.

Sky islands (SI) are globally distributed isolated mountain habitats surrounded by lowlands or habitats outside of the range of species’ thermal tolerance, serving as a barrier to species dispersal and migration^12,13^ (Fig. 1). While climatic variation during the Pleistocene allowed for panmixia and species migration and dispersal up and down slope, ultimately, warming and drying after the Last Glacial Maximum (LGM) drove many populations back up slope, where they have remained isolated for 10–18,000 years^12,14–17^. In fact, the distributions of many species on SI can be explained by lowland extinctions after the LGM^17,18^. With Anthropogenic climate change and continuing up-slope range contractions, the potential for gene flow between small populations atop SI has further declined^19–21^. Conversely, adjacent mountain chains (MC) are vast mountainous regions whereby multiple large populations may exist, interact, and migrate between contiguous habitat corridors^22–25^. Owing to more habitat on MC, populations experience higher levels of gene flow and genetic diversity, as well as more climatically buffered habitat suitable for migration and range shifts. Overall, populations across SI and MC have experienced long-term differences in climates that have continued to diverge under Anthropogenic climate change.

Sky islands have been used extensively as laboratories for studying evolutionary ecology—specifically, patterns of diversity and allopatric speciation—in response to geographic isolation and post-Pleistocene demographic shifts^12,14,15^. Previous work has shown that isolation of SI by environment and by distance has resulted in genetic divergence, shifts in species interactions, reduction in genetic variation, and rapid speciation on SI^17,26–33^. These studies show the important roles of reduced gene flow, high environmental pressures, genetic drift, and historical population demographics that have shaped populations isolated atop SI since the Pleistocene^17,34,35^. However, their use as a tool for examining hypotheses specifically related to past or contemporary climate change is minimal. Most research has examined only single SI or clusters of SI within a circumscribed geographic region, and very few incorporate climatic differences into statistical models. Comparative studies of SI relative to adjacent, continuous MC (i.e., the SI-MC comparison) can directly examine trait evolution and evolutionary mechanisms important for population persistence and ecosystem function in response to past and current climate change.

We use the SI-MC comparison to determine if a long-lived tree species has adapted in response to climate change and isolation. Using clonal replicates of trees collected from SI and adjacent MC in riparian areas in the western U.S., we examined intra-specific, population-level divergence to test how vegetative reproductive and productivity traits have responded to long-term climate change and isolation. Under common garden conditions, we examined how long-term climatic variation in temperature and precipitation has altered cloning (i.e., asexual or vegetative reproduction) and aboveground biomass production (i.e., terrestrial productivity) in contemporary populations of the dominant foundation species *Populus angustifolia*^36^. We tested the hypothesis that *P. angustifolia* populations on hotter, drier SI have phenotypically diverged from populations on MC. Specifically, based on previous research that reports adaptive phenotypic traits driven by landscape-level climatic variation^1^, we hypothesized that with increased climatic pressures on SI (i.e., heat and drought), *P. angustifolia* populations grown in a common garden will have greater rates of cloning and aboveground biomass than populations on MC, and that these two traits are correlated across the entire study region (Hypothesis 1). Lastly, we tested the hypothesis that any phenotypic variation is driven by climate-induced natural selection (Hypothesis 2).

## Results

**Figure 2:**
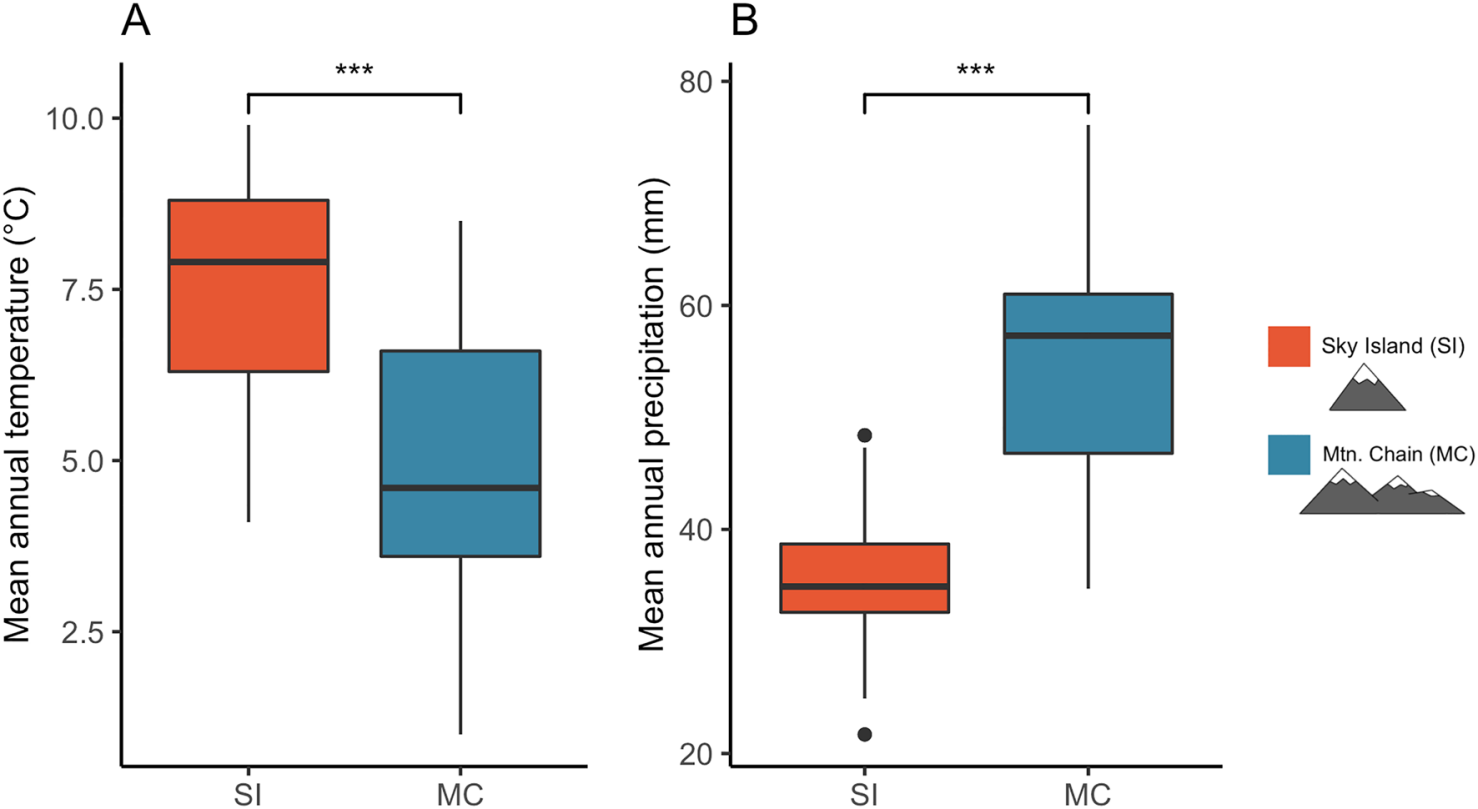
The climate is significantly different between sky islands (SI) and mountain chains (MC). (**A**) Mean annual temperature (degrees Celsius) is 35% higher on SI than on MC. **(B**) Mean annual precipitation (millimeters) is 53% lower on SI than MC. Asterisks denote a significant difference (*p < 0.05).

**Figure 3:**
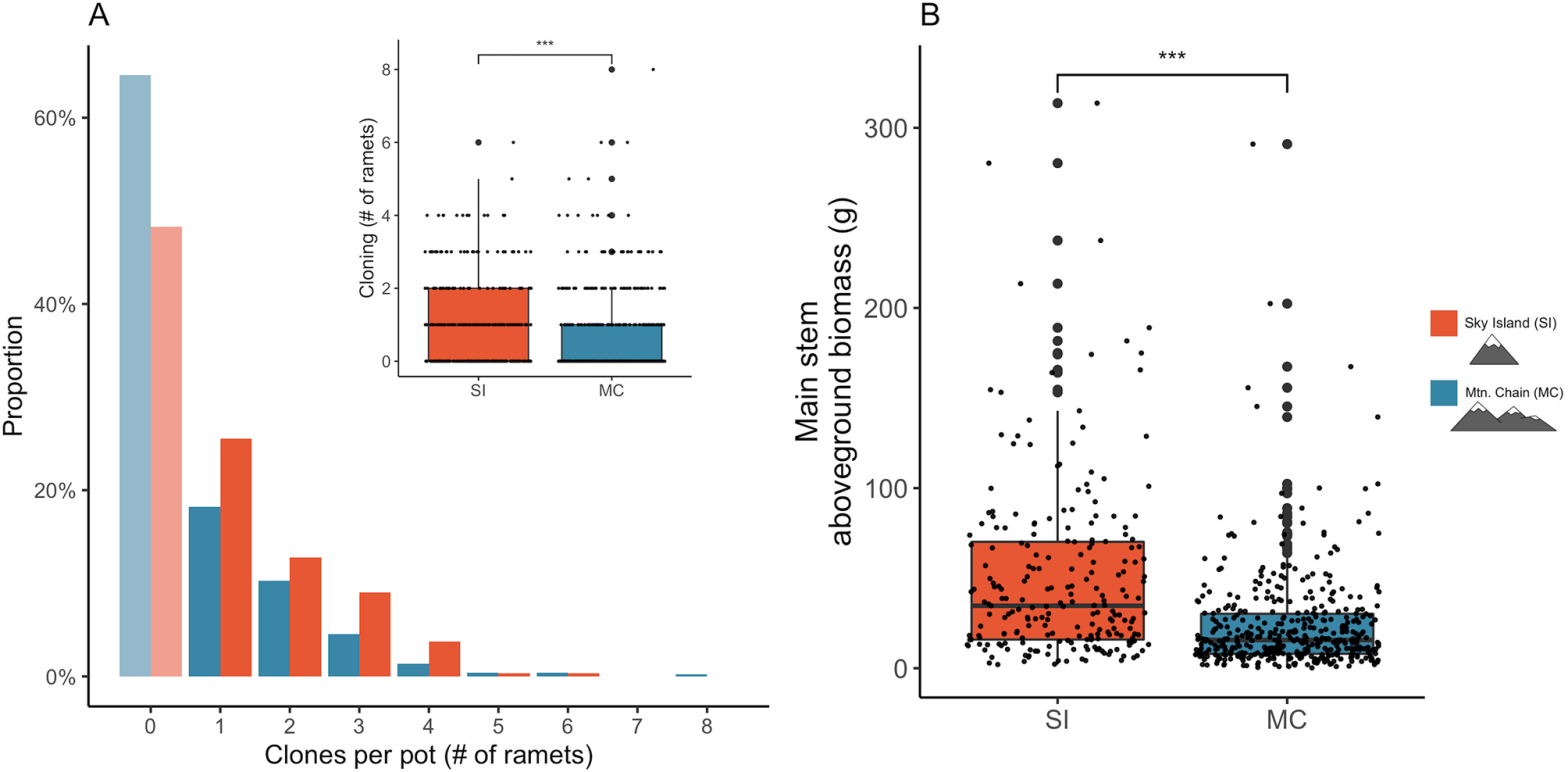
Functional traits of *Populus angustifolia* diverge between sky islands (SI) and mountain chains (MC) when grown in a common environment. (**A**) The mean number of clonal ramets and (**B**) aboveground biomass are significantly higher on SI than MC. Panel (**A**) shows the normalized mass of cloning ramets (#) on SI (orange) and on MC (blue), separately. Each orange bar shows the proportion of ramets from trees from SI with a given cloning ramet number (x-axis), blue bars correspond to the same for MC; all bars in each color add up to 1. The inset is a box and whisker plot representative of these data. In each box and whisker plot, black circles denote genotype replicates (n = 193 genotypes on MC; n = 57 genotypes on SI). Asterisks denote a significant difference (*p < 0.05).

Consistent with Hypothesis 1 and the strong environmental differences between SI and adjacent MC, we found that common garden populations from SI converge in functional traits important to population persistence under stressful environmental conditions (Fig. 2). Specifically, common garden populations from SI had 34% higher rates of cloning (number of ramets) and produced 52% more aboveground biomass (g) than those from MC (Fig. 3; Table 1). Additionally, we found a weak but significant positive correlation (R^2^ = 0.022, *p*-value = 0.05016*) between cloning and aboveground biomass, suggesting a possible genetic linkage between the gene complexes responsible for the two traits (Supplementary Fig. S1).

**Table 1:** Reproductive and productivity traits (cloning and aboveground biomass, respectively) from sky island populations (SI) have diverged from mountain chain populations (MC).

Response	Fixed effects		Chi-square test		Analysis of variance		
	Std. Error	Z-score	χ^2^	Pr (> χ^2^)	SS	MS	F value
Cloning	0.227	2.081	4.331	0.037*	4.340	4.340	4.340
Biomass	0.197	3.368	11.347	0.001*	8.310	8.310	11.347

Data refer to summary statistics for generalized linear mixed-effects and linear mixed-effects models, respectively, where cloning and biomass were predicted by SI and MC as a fixed effect, with watershed/genotype as a nested random effect. Asterisks indicate quantitative trait differences that are significantly different from zero (*p < 0.05).

In partial support of Hypothesis 2, we found that both natural selection and genetic drift were responsible for patterns of genetic divergence for aboveground biomass and cloning, respectively. Based on previously established methods for determining the evolutionary role of natural selection versus genetic drift, we used a previously published ANCOVA approach that incorporates neutral genetic microsatellites as a covariate into the phenotypic models used in Hypothesis 1 to account for population genetic structure^37^. Specifically, we performed a principal components analysis (PCA) on the z-scores of the microsatellite associated with individual genotypes and incorporated the first two principal component axes (PC1 and PC2) into the phenotypic models. Incorporating genetic markers in the trait models to detect any remaining significance attributed to mountain class (i.e., SI or MC) allows us to interpret any observed quantitative trait differences as a consequence of natural selection or genetic drift induced by mountain class. When microsatellite data were included in the phenotypic models (Table 1), we found that the differences in aboveground biomass due to mountain class remained significantly different even after accounting for neutral genetic variation (Table 2). This same framework showed that when neutral genetic variation is included in the cloning model, the variation attributed to mountain class is no longer significant (Table 2). Using Akaike Information Criterion (AIC), we found that both models performed best when the microsatellite data were included, suggesting that genetic drift is present in both populations, but does not significantly explain the phenotypic variation in aboveground biomass (Table S1). Overall, we conclude that natural selection from climate on SI is the driving evolutionary force behind variation in aboveground biomass, and genetic drift from isolation on SI is the driving force for variation in cloning.

**Table 2:** Convergent trait variation is driven by natural selection for aboveground biomass and genetic drift for cloning.

Factors	χ^2^	Pr (> χ^2^)
Response: cloning		
Mtnclass	0.723	0.395
PC1	0.091	0.763
PC2	0.026	0.873
Response: aboveground biomass		
Mtnclass	33.531	7.013e−09*
PC1	1.729	0.189
PC2	0.582	0.445

Data refer to summary statistics for mixed-effects models where cloning and aboveground biomass response variables were predicted by mountain class (“Mtnclass”: SI or MC) and microsatellite principal components axes (PC1 and PC2), with watershed/genotype as a nested random effect. Asterisks indicate differences that are significantly different from zero (*p < 0.05).

## Discussion

Overall, we show phenotypic trait divergence across the natural range of a long-lived tree species driven by ongoing population demographic and climatic differences since the end of the Pleistocene across the SI-MC comparison. Asexual, vegetative reproduction via cloning allows a genet to spatially “scavenge” for resources across the landscape and persist in environments post-perturbation or under stress^35,38–41^. Adaptation toward both greater vegetative reproduction and increased productivity (i.e., aboveground biomass) in higher-stress populations of *P. angustifolia* on SI may indicate that phenotypic trade-offs between reproduction and growth common in high-stress environments^42–45^ are not as essential to the survival of this species^46^. An alternative hypothesis for this pattern is that natural selection is acting on the fitness trait of aboveground biomass. Because cloning and aboveground biomass are positively correlated, high-aboveground biomass trees also produce more clones. Regardless, increases in both of these traits in warmer and drier populations on SI is representative of increased carbon sequestration that influences overall ecosystem productivity^47–49^. Taken together, the altered reproductive and productivity traits that we document indicate that convergent evolution among SI could lead to predictable ecosystem-level consequences driven by climatic variation relative to MC, irrespective of the evolutionary mechanism at play. Further, across the SI-MC comparison, cloning and aboveground biomass were significantly correlated, suggesting genetic linkage between the two traits that may be influenced by environmental variation. Due to its value as a bioenergy crop, much research has gone into identifying genetic controls on aboveground biomass in the genus *Populus*^50–54^. However, no research to our knowledge has attempted to tackle the genetic underpinnings of cloning as a reproductive strategy in *Populus*. Thus, further research is needed to elucidate the genetic and functional significance underlying the correlation between cloning and aboveground biomass.

Contemporary climate change is projected to increase temperatures and the prevalence of severe drought on a global scale, especially in the western U.S.^55,56^. Numerous studies suggest that plant species will likely respond to the stress caused by climate change by implementing shifts in their geographic ranges^57–61^. However, relying on bioclimatic envelopes to predict and model future species’ distributions in response to climate change has largely underestimated the adaptive capacities of long-lived species through phenotypic plasticity or rapid evolution^62,63^. One phenotypically adaptive mechanism by which populations may persist under rapid climate change is through evolutionary shifts in cloning (i.e., asexual regeneration) and overall biomass production (i.e., productivity). Evidence of trait convergence in long-lived tree species demonstrates the empirical utility of the SI-MC comparison as a natural laboratory and predictive framework for studying evolution in response to climate change in natural populations.

The SI-MC comparison should allow for a greater understanding of the genetic basis to phenotypes that promote population-level persistence in stressful environments and determine the ecological and evolutionary mechanisms (i.e., selection or drift) that drive ecosystems to persist globally^35,64^. The global breadth of the SI-MC comparison is ripe for studies that examine the genetic basis by which populations persist or perish across a fragmented and vulnerable contemporary landscape. For example, SI and MC can be found across the entire natural range of *P. angustifolia* from northern Mexico to Alberta, Canada, and are present in all three genetic provenances identified for the species^36^. Moreover, SI and MC are found on almost every continent and similar comparisons are possible across many species of plants, animals and microorganisms, providing comparable opportunities to examine evolution in response to climate and isolation around the globe.

Previous work detailing the evolutionary responses of species isolated atop individual or adjacent SI has shown the dramatic impact that isolation and climatic exposure on SI can have on a wide variety of taxa^26,28,30,65–67^. We suggest that the study of SI may be made more robust by studying geographically adjacent MC comparatively to address evolution in direct response to climate change, as opposed to other approaches that infer evolution in response to climate variation. The climatic histories and demographic structure (i.e., low levels of gene flow and limited dispersal, and high selective pressures and potential for genetic drift) commonly found on SI, relative to MC, make SI-MC comparisons ideal for future climate change studies across the globe and will allow for direct tests of the role of climate change. Using the SI-MC comparison across a large geographic scale to understand population- and community-level responses to climate change will allow for a better understanding and more robust predictions of climate-driven evolution, and subsequent ecosystem-level responses, across the globe.

## Methods

### Study species and site selection

*Populus angustifolia* James (Salicaceae) is a native high-elevation, foundation tree species dominant in riparian areas (900–3500 m) across the intermountain western U.S.^68^. Throughout its natural range, *P. angustifolia* forms distinct genetic populations, largely driven by reduced gene flow resulting from variation in the intermountain landscape^69^. The fragmented contemporary range of *P. angustifolia* reflects historical bottlenecks driven by Pleistocene glacial cycles, as well as contemporary climate change^18,69^. *Populus angustifolia* extends northwards from northern Mexico into southern Alberta, Canada; a geographic region also characterized by the presence of SI^12,69^. Sky islands are isolated mountain habitats surrounded by desert lowlands or habitats outside of the range of species’ thermal tolerance, serving as a barrier to species dispersal and migration^12,13^. Conversely, populations of trees from the continuous MC throughout the natural range of *P. angustifolia* are not dispersal-limited, as the riparian habitat between the high-elevation zones on MC is climatically suitable for within-population windborne seed dispersal and migration. *Populus angustifolia* exhibits both asexual and sexual reproductive strategies, mostly by cloning and pollen/seed dispersal by wind and water^70,71^. Due to the obligate riparian nature of *P. angustifolia*, populations on SI used in this study constitute distinct populations, devoid of the potential for any significant gene flow^36^. Isolated SI across the landscape, paired with multiple adjacent MC, serve as natural replication for our study^72^. In May and June of 2012, 8 watershed populations (3 SI: Indian Creek “IC”, UT; Lexington Creek “LEX”, NV; Great Basin “GBS”, NV. 5 MC: Dolores River “DOL”, CO; Logan River “LOG”, UT; Ogden Canyon “OGC”, UT; San Miguel River “SMIG”, CO; Weber River “WR”, UT) of *P. angustifolia* were surveyed (Fig. 1a). Georeferenced climate data for mean annual temperature and mean annual precipitation were collected for each tree from WorldClim^73^. In the field, 1350 genotype replicates (n = 193 genotypes from MC; n = 57 genotypes from SI) were georeferenced and sampled and 10 terminal branch cuttings (~ 20 cm long) were collected from each tree. The cuttings were initially established in general potting soil (equal parts vermiculite, perlite, and peat) for 4 months^1^. Surviving cuttings were replanted in 6.4 × 36 cm plastic pots and grown under identical ambient common garden conditions at the University of Tennessee Knoxville greenhouse.

### Greenhouse common garden and quantitative trait measurements

To understand how *P. angustifolia* populations on SI have diverged in stress-adaptive functional traits (Hypothesis 1), cuttings were grown in the University of Tennessee Knoxville greenhouse. All plants in the common garden experienced similar environmental conditions: they were watered every 2–3 days under near-ambient seasonal temperature conditions. This setup allowed for the isolation of the genetic basis to trait variation. In 2012, aboveground biomass (g) was measured from the main stem of each replicated tree genotype using the following allometric equation: Aboveground biomass (g) = (stem volume (mm^3^) × 0.41899) – 2.40137, whereby stem volume (mm^3^) = π (0.5 × basal stem diameter)^2^ × plant height (mm)^74^. In 2016, cloning was quantified from all surviving greenhouse trees by counting the number of ramets present within each pot. Cloning was measured in 2016 as this was the year that cuttings began to produce ramets.

### Molecular genetic analyses

We used microsatellite data to resolve the observed evolutionary patterns in quantitative traits, identify individual genotypes of each tree, and determine population genetic divergence in the study areas. In the field, leaves were collected from each genotype and were dried in a 70 °C oven for 48 h, finely ground using a SPEX SamplePrep 8000D Dual Mixer (SPEX SamplePrep, Metuchen, NJ, USA); genomic DNA (gDNA) was extracted using a Qiagen DNeasy Plant Mini Kit (Qiagen, Valencia, CA, USA) following manufacturer’s protocols. To reduce PCR inhibition, the gDNA samples were diluted at 1:10 (1-part gDNA, 9 parts molecular-grade water). We used nine microsatellite markers to determine multi-locus genotypes for each tree^75,76^. PCR amplification and electrophoresis were executed using previously published techniques^1^. To control for clonal genotypes, we used the “multilocus matching” function in GenAlEx v.6.4^77,78^ to identify samples with 100% microsatellite loci similarity.

### Statistical analyses

To confirm previously reported climatic differences across the SI-MC comparison, we employed linear mixed-effects models for mean annual temperature (MAT) and mean annual precipitation (MAP) response variables, separately, with mountain class (SI or MC) as the fixed effect and watershed population as a random effect (“lme4” package, R^79^). To address the contribution of elevation to any observed population-level variation and to eliminate the alternative hypothesis that ranges have already shifted, we conducted a linear mixed-effects model with elevation as the response variable, mountain class (SI or MC) as the fixed effect, and watershed as a random effect. Subsequent ANOVAs were conducted for all models.

To address the hypothesis that isolated populations of SI have diverged in stress-adaptive functional traits, relative to MC (Hypothesis 1), we used a generalized linear mixed-effects model with a Poisson log link function for the cloning response variable, with mountain class (SI or MC) as a fixed effect, watershed/genotype as a nested random effect. This model was selected to account for the right-skewed cloning count data with a Poisson distribution. For the aboveground biomass response variable, we used a linear mixed-effects model with mountain class (SI or MC) as a fixed effect and watershed/genotype as a nested random effect. Aboveground biomass was log-transformed for normality. For both models in Hypothesis 1, a nested random effect of watershed/genotype was chosen to account for variation at each level (i.e., watershed, genotype). This procedure allowed for a more accurate assessment of any observed SI-MC effect and is a commonly used practice in classical studies in evolutionary ecology with nested design^80^. Subsequent ANOVAs were performed on the cloning and aboveground biomass models to determine significance. A two-sided Pearson’s product-moment trait correlation was used to test the hypothesized quantitative trait linkage between cloning and aboveground biomass across the SI-MC comparison. 

To examine the mechanisms of evolution (i.e., natural selection or genetic drift) driving trait divergence (Hypothesis 2), we used an ANCOVA approach that incorporated neutral microsatellite data into the mixed-effects models employed for Hypothesis 1^37^. To reduce dimensionality and account for loci collinearity, we first performed a principal component analysis (PCA) ordination on the z-scores of the microsatellite data. We then extracted the main principal components that explained the majority of the microsatellite variance (PC1 and PC2). The principal component axes were then added to the quantitative trait mixed-effects models described for Hypothesis 1. To test model fit for each trait, models employed in Hypothesis 1 without microsatellite PC axes were used as null models and were run in parallel with the alternate models that included microsatellite PC axes. Akaike Information Criterion (AIC) estimates were compared between each. For each trait, models containing PC1 and PC2 performed better than the null models without the PC axes, suggesting that genetic drift is present within both populations. For Hypothesis 2, if the observed variation in cloning and aboveground biomass across the SI-MC comparison seen in Hypothesis 1 remained significant after adding the microsatellite PC axes to the models, the evolutionary process driving the trait variation is consistent with adaptive trait differentiation (i.e., natural selection). Conversely, if the observed trait variation becomes insignificant after incorporating the microsatellite ordination axes, neutral and demographic processes (i.e., genetic drift) are more likely responsible for trait variation across the landscape^37,81^. For all hypotheses, statistical significance (*p* < 0.05) was determined by Anova Type II Wald chi-square tests for each model (“Anova” function, “car” package, R^82^.

## Supplementary Information

Supplementary Information 1.

Supplementary Information 2.

Supplementary Information 3.

Supplementary Information 4.

Supplementary Information 5.

## Data Availability

Deidentified data analyzed during this study are included in the Supplementary Information. Full datasets used and analyzed during the current study are available from the corresponding author on reasonable request.
