# Supplementary material for: Climate-driven convergent evolution in riparian ecosystems on sky islands
Source: Sci Rep. 2023 Feb 16;13:2817. doi: 10.1038/s41598-023-29564-2 (PMC9935884; doi:10.1038/s41598-023-29564-2)
Supplement: Supplementary file 5 — Supplementary Information 5. [file 41598_2023_29564_MOESM5_ESM.docx]

Climate-driven convergent evolution in riparian ecosystems on sky islands

**SJ Love^1,^*, JA Schweitzer^1^, JK Bailey^1^**

1. Department of Ecology and Evolutionary Biology, University of Tennessee, Knoxville, Tennessee, 37996, USA

*Corresponding author:

Email: [sjaynelove@gmail.com](mailto:sjaynelove@gmail.com)

Current address: Department of Ecology and Evolutionary Biology, University of Tennessee, Knoxville, Dabney Hall, 1416 Circle Dr, Knoxville, TN 37996

# Supplementary information

# Tables

| Supplementary Table S1 \| Increased performance of cloning and aboveground biomass trait models with neutral genetic markers. | |
| --- | --- |
| **Model** | **AIC*** |
| **Response: Cloning﻿^†^** |  |
| *Null, without PC axis* |  |
| glmer (Cloning ~ Mtnclass + (1\|Watershed/Genotype), family = Poisson (link = log)) | 1993.992 |
| *Alternative, with PC axis* |  |
| glmer (Cloning ~ Mtnclass + PC1 + PC2 + (1\|Watershed/Genotype), family = Poisson (link = log)) | 861.278 |
| **Response: Aboveground (Abg.) biomass** |  |
| *Null, without PC axis* |  |
| lmer (Abg. biomass ~ Mtnclass + (1\|Watershed/Genotype)) | 1792.908 |
| *Alternative, with PC axis* |  |
| lmer (Abg. biomass ~ Mtnclass + PC1 + PC2 + (1\|Watershed/Genotype)) | 639.045 |
| ^*^Model fit using Akaike Information Criterion (AIC). ^†^Cloning models utilize a generalized linear mixed model with a Poisson log link function to best fit the cloning count data. Mtnclass is a categorical variable denoting sky island or mountain chain. | |

# Figures

Supplementary Figure S1 | Cloning and aboveground biomass functional traits of *Populus angustifolia* are significantly positively correlated. Cloning (number of clones per genetic replicate) and aboveground biomass (grams; log-transformed) are significantly, positively, correlated (r=0.15; p=0.05016*).


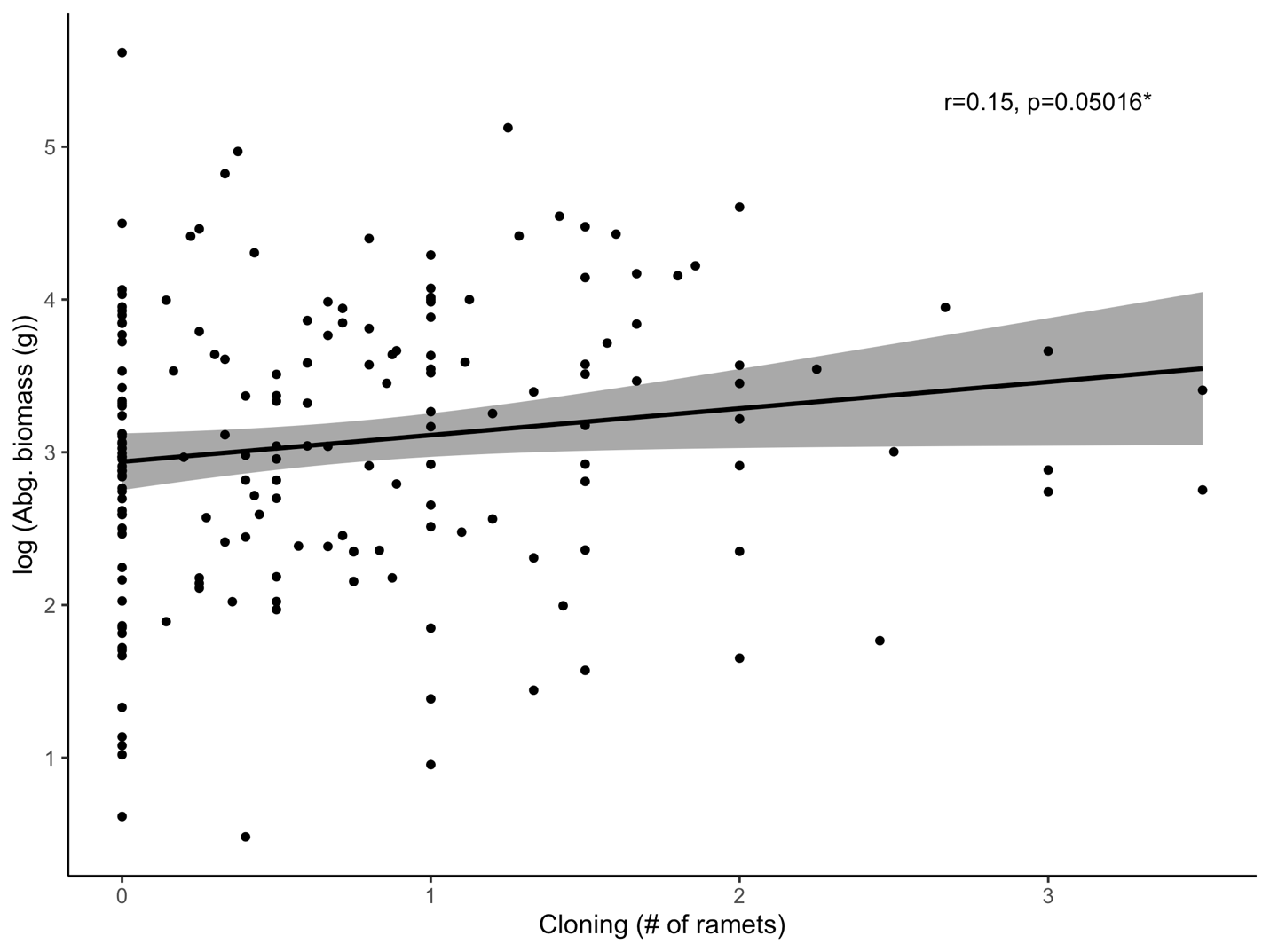


**Supplementary Figure S1**
